# Effect of Formononetin on Mechanical Properties and Chemical Composition of Bones in Rats with Ovariectomy-Induced Osteoporosis

**DOI:** 10.1155/2013/457052

**Published:** 2013-05-12

**Authors:** Ilona Kaczmarczyk-Sedlak, Weronika Wojnar, Maria Zych, Ewa Ozimina-Kamińska, Joanna Taranowicz, Agata Siwek

**Affiliations:** Department of Pharmacognosy and Phytochemistry, Medical University of Silesia, Poniatowskiego 15, 40-055 Katowice, Poland

## Abstract

Formononetin is a naturally occurring isoflavone, which can be found in low concentrations in many dietary products, but the greatest sources of this substance are *Astragalus membranaceus, Trifolium pratense, Glycyrrhiza glabra,* and *Pueraria lobata*, which all belong to Fabaceae family. Due to its structural similarity to 17**β**-estradiol, it can mimic estradiol's effect and therefore is considered as a “phytoestrogen.” The aim of this study was to examine the effect of formononetin on mechanical properties and chemical composition of bones in rats with ovariectomy-induced osteoporosis. 12-week-old female rats were divided into 4 groups: sham-operated, ovariectomized, ovariectomized treated with estradiol (0.2 mg/kg) and ovariectomized treated with formononetin (10 mg/kg). Analyzed substances were administered orally for 4 weeks. Ovariectomy caused osteoporotic changes, which can be observed in bone biomechanical features (decrease of maximum load and fracture load and increase of displacements for maximum and fracture loads) and bone chemical composition (increase of water and organic fraction content, while a decrease of minerals takes place). Supplementation with formononetin resulted in slightly enhanced bone mechanical properties and bone chemistry improvement (significantly lower water content and insignificantly higher mineral fraction content). To summarize, administration of formononetin to ovariectomized rats shows beneficial effect on bone biomechanical features and chemistry; thus, it can prevent osteoporosis development.

## 1. Introduction

Estrogen, as an antiresorptive agent, interacts via estrogen receptors *α* and *β* with osteoblasts as well as inhibits osteoclastogenesis and prevents bone loss. Thus, in women during menopause, when level of estrogen decreases, osteoporosis may occur, which could lead to pathologic fractures [1–4]. In order to reduce menopausal symptoms, including osteoporosis, hormone replacement therapy (HRT) has been used. This treatment, however, has also been proven to have negative side effects—it could lead to breast cancer, abnormal uterus bleedings, or cardiovascular diseases in postmenopausal women [5, 6]. To overcome adverse effects of HRT, an alternative therapy became necessary, and the promising substances for this purpose are phytoestrogens [7, 8]. Phytoestrogens are polyphenolic, nonsteroidal secondary plant metabolites which can be divided into the following classes: flavonoids, especially isoflavonoids, coumestans, lignans, and mycoestrogens. Due to their structural similarity to 17*β*-estradiol, they may bind to estrogen receptors and mimic the effect of estradiol, but their activity is 10^−2^–10^−3^-fold lower [9–11]. Among all known phytoestrogens, isoflavones are widely studied. Genistein and equol exhibit more potent estrogenic effects than other substances from this group [12, 13]. Another isoflavone that shows estrogenic activity is formononetin. Formononetin—7-hydroxy-3(4-methoxyphenyl)chromone, (C_16_H_12_O_4_)—is the major compound of the *Astragalus membranaceus *and* Astragalus mongholicus *root, but it could also be found in leaves and flowers of *Trifolium pratense*, roots of *Glycyrrhiza glabra* as well as *Glycyrrhiza uralensis*, and in root of kudzu—*Pueraria lobata* [14–18]. This isoflavonoid could also occur in dietary products like beans, carrot, cauliflower, green peas, iceberg lettuce, or red potatoes [19]. Formononetin has been proven to show antimicrobial activity towards *Giardia lamblia* [16], acting as both an antioxidant [20] and as a neuroprotective agent in Alzheimer's disease [21]. This isoflavonoid has also been reported to have positive effect on osteoarthritis [15] and promote early fracture healing through stimulating angiogenesis [22]. What is more, it can be used in cardiovascular diseases as vasorelaxant [14] as well as in the prevention of myocardial infraction due to its cardioprotective activity [23]. Formononetin also possesses hypolipidemic properties, mammary gland proliferation function, and antitumor effect in colon and breast cancer [17, 24–26]. Estrogenic effect of formononetin has also been reported. This isoflavone has structural similarity with 17*β*-estradiol; thus, it can bind to estrogen receptors, for example, in bone tissue [13, 20, 23]. Up till now, there have been no reports about the effect of formononetin on chemical composition and mechanical properties of bones.

Due to the fact that the analysis of bone mechanical features and their resistance to fractures is impossible to be conducted on human, a laboratory animal model was developed. The best animal model for this purpose is ovariectomy, where estrogen deficiency occurs; thus, osteoporotic changes in animal skeletal system can be observed. Osteoporotic changes in ovariectomized (OVX) rats are similar to those in postmenopausal women; therefore, OVX rats are good models for postmenopausal osteoporosis [27].

In our study, the effects of formononetin on chemical composition and mechanical properties of bones in ovariectomized rats were investigated for the first time.

## 2. Materials and Methods

The experiment was conducted on the three-month-old female Wistar rats which were fed a standard laboratory chow *ad libitum*. Since the day preceding the experiment, the standard chow was replaced with chow containing no soybean. Rats were provided by the Centre of Experimental Medicine at the Medical University of Silesia. The research was carried out with the approval of the Local Ethics Commission in Katowice.

Rats were divided into four groups (*n* = 7): the control group of sham-operated, vehicle-treated rats (SHAM), the control group of ovariectomized, vehicle-treated rats (OVX), the ovariectomized rats receiving estradiol at a dose of 0.2 mg/kg *po* (OVX + ES), and the ovariectomized rats receiving formononetin at a dose of 10 mg/kg *po* (OVX + FRM). The analyzed substances were administered for four weeks.

The body weight of the rats was determined both on the first day of the research and after four weeks following the administration of the substances (body mass gain was also controlled during the whole experiment). Seven days before administering estradiol and formononetin, the bilateral ovariectomy and the sham surgery in general anesthesia induced by the mixture of ketamine and xylazine i.p. were performed. After four weeks of drugs administration, all rats were sacrificed with the use of general anesthesia induced by ketamine and xylazine. Each of the rats had the right and left tibia and the right and left femur excised, as well as the L-4 vertebra, uterus, and thymus. The mass of femur, tibia, and L-4 vertebra was presented as the ratio of their mass per body mass determined after four weeks of the experiment.

### 2.1. The Analysis of the Mechanical Properties of the Bones

Testing of the bone mechanical properties of the left femoral diaphysis, right tibial proximal metaphysis, and right femoral neck was performed using the Instron apparatus, model 3342 500 N. Results were studied by using the software Bluehill 2, version 2.14.

The femur was placed horizontally in the testing apparatus, and the bending test with three-point loading was applied, as previously described [28]. The load was applied perpendicularly to the long axis of the femur, at its midlength. The distance between the supporting points was 16 mm. The load was applied perpendicularly to the long axis of the femur at the midlength of the bone (displacement rate of 0.01 mm/s, sampling rate of 100 Hz). During the experiment, the following parameters for femoral diaphysis were measured: the maximum load, the fracture load, displacement for maximum load, and displacement for fracture load. Young's modulus was also evaluated.

Assessment of the mechanical properties of tibial proximal metaphysis was performed according to the method of Stürmer et al. [28, 29], using the three-point bending test. After applying the preload of 1 N, the load test was performed with the displacement rate of 0.01 mm/s. During the experiment, the following parameters for tibial metaphysis were measured: the maximum load, the fracture load, displacement for maximum load, and displacement for fracture load. Young's modulus was also determined.

The mechanical properties of the femoral neck were analyzed by performing a compression test [28, 30]. Right femoral bone was cut at the midlength. Obtained bone segment was affixed to the holes in the methacrylate plates using the epoxy resin. The load was applied to the femoral head, parallel to the femoral long axis. The mechanical compression test with the displacement rate of 0.01 mm/s was performed, after preload of 1 N. During the experiment, the maximum load affecting the femoral neck was determined.

### 2.2. Assessment of the Chemical Content of the Bones

The assessment of the chemical content involved the calculation of the water, organic substances, and mineral substances content as well as content of calcium and phosphorus in left femur and left tibia (after bone mechanical properties analysis) and L-4 vertebra.

In order to determine the water content, the tibia, femur, and L-4 vertebra were lyophilized in the lyophilizer Labconco FreeZone 6 for five days (temperature: −53°C, pressure: 0.03 mBa) and then weighed. The difference between the bone mass obtained directly after the isolation and the bone mass determined after lyophilization corresponds to the water content.

The lyophilized bones were mineralized for 48 hours in the muffle furnace type LG/11/C6 produced by Nabertherm. After mineralization, bones were weighed. On the basis of the difference between bone mass determined after lyophilization and bone mass after mineralization, the content of the organic substances was estimated.

The bone ash obtained during the mineralization process comprised the mass of the mineral substances.

The content of water, organic, and mineral substances was presented as the ratio of water, organic, and mineral substances mass per 100 mg of the bone mass after isolation. Mineralized bones were dissolved in 6 M HCI, and diluted in distilled water, and then calcium and phosphorus content was determined by the colorimetrical method, using the kit Pointe Scientific, Inc. The content of calcium and phosphorus in the analyzed bones was presented as the ratio of the quantity of calcium and phosphorus per 100 mg of mineral substances.

### 2.3. Statistical Analysis

The results obtained during the tests were presented as arithmetic means ± SEM. The results were formulated by one-way ANOVA followed by Student's *t*-test to detect the intergroup differences. The differences were considered to be statistically significant if *P* < 0.05.

## 3. Results

### 3.1. Effects of Ovariectomy-Induced Estrogen Deficiency in Rats

After four weeks of the experiment, in the ovariectomized rats (OVX), statistically significant body mass gain (by 57.6%) was noted, as well as the uterus mass loss (statistically significant by 76.0%) and the thymus mass gain (statistically significant by 74.3%), as compared to the sham-operated control group of rats (SHAM) (Table 1).

Mass of the femoral bone (statistically significant by 10.1%), tibia (statistically significant by 9.9%), and L-4 vertebra (by 14.1%) decreased in OVX rats when compared to the SHAM group. In comparison with the SHAM group, the femoral and tibial bone lengths of OVX rats were not significantly affected, while the diameter of the femur (by 5.3%) and tibia (statistically significantly by 7.4%) decreased (Table 2).

In the OVX group, maximum load and fracture load decreased (by 6.2% and 10.0%, resp.), while displacement for both maximum load and fracture load increased (resp., by 8.3% and 18.0%), as compared to the SHAM group. Young's modulus value in the femoral diaphysis decreased (by 6.1%) when compared to the SHAM group. There was a statistically significant decrease in maximum load (by 51.0%) and fracture load (by 54.5%) of the tibia metaphysis in comparison to the SHAM group. In comparison to the SHAM group, in OVX group, displacement for maximum load in tibial metaphysis was not significantly changed, while displacement for fracture load increased (by 14.9%). Young's modulus value decreased statistically significantly (by 50.9%) when confronted with the SHAM group. In the OVX group, the maximum load applied to the femoral neck was decreased (by 13.1%), as compared to the SHAM group (Table 3).

Statistically significant increase of water content in femur, tibia, and L-4 vertebra (by 15.2%, 15.8%, and 17.4%, resp.), as well as statistically significant increase of the organic substances content in femur (by 16.2%), tibia (by 16.7%), and L-4 vertebra (by 25.2%) was noted in OVX group as compared to the SHAM group. Furthermore, a decrease of mineral content in femur (statistically significant by 15.9%), tibia (by 18.0%), and L-4 vertebra (statistically significant by 26.8%) in OVX group in comparison to the SHAM group was observed. The content of calcium in femur, tibia, and L-4 vertebra was lowered (by 10.8%, 9.2%, and 8.7%, resp.), whereas the content of phosphorus was not changed when compared to the SHAM group (Table 4).

### 3.2. Effects of Estradiol Administration to Ovariectomized Rats

Administration of estradiol to ovariectomized rats (OVX + ES) resulted in a statistically significant reduction of body mass gain (by 32.6%), increased uterus mass (by 96.9%), and a decrease of the thymus mass (by 9.8%), in comparison with the control group OVX (Table 1). An increased mass of the femur, tibia, and L-4 vertebra (by 6.0%, 3.9%, and 11.5%, resp.) was noted in OVX + ES group as compared to the OVX rats. The length of the femoral and tibial bone in OVX + ES rats was not changed, while the diameter of both the femur and tibia increased (resp., by 2.1% and 3.8%) in comparison to the OVX group (Table 2).

There were changes in bone mechanical properties in OVX + ES rats observed: an increase in the maximum load and the fracture load in femoral diaphysis (by 5.7% and 11.2%, resp.), while displacement for maximum load and displacement for fracture load in femoral diaphysis were decreased (by 10.8% and 16.7%, resp.). Young's modulus value in femoral diaphysis increased (by 12.5%), as compared to the OVX group. In comparison to the OVX rats, the maximum load and the fracture load in tibial metaphysis of OVX + ES group increased (by 34.8% and statistically significantly by 49.6%, resp.), displacement for maximum load was not changed, while displacement for fracture load decreased (by 8.6%). Value of Young's modulus increased (by 8.9%) in OVX + ES group when compared to the OVX group. The administration of estradiol affected the maximal load in femoral neck, which increased by 11.5%, in comparison with the OVX group (Table 3).

In femur of the OVX + ES rats, there was a decrease of water (statistically significantly by 8.1%) and the organic fraction content (statistically significantly by 16.4%), while the content of minerals (statistically significantly by 16.5%), and calcium (by 8.7%) increased, when compared to the OVX group. In tibia, a decrease of water (statistically significant by 10.7%) and organic substances (statistically significant by 18.0%) was observed, while an increase of minerals (statistically significant by 23.1%) and the calcium content (by 12.8%) was noted in OVX + ES group when confronted with the OVX rats. There was a decrease in the water (by 13.9%) and organic fraction content (statistically significantly by 17.6%) in the L-4 vertebra of OVX + ES group, whereas the content of mineral substances (statistically significantly by 32.3%) and calcium (by 3.5%) increased. The phosphorus content was not changed in any of the analyzed bones when compared to the OVX group (Table 4).

### 3.3. Effects of Formononetin Administration on Ovariectomized Rats

The administration of formononetin to ovariectomized rats (OVX + FRM) did not cause any changes in the thymus and uterus mass as well as in the body mass gain as compared to the OVX group (Table 1).

In OVX + FRM rats, an increase in the mass of the femur, tibia, and L-4 vertebra (by 4.4%, 2.2%, and 9.8%, resp.) was observed. The length of femur and tibia was not changed, while there was an increase in the femoral bone diameter (by 3.5%) and tibial bone diameter (by 4.7%), in comparison with the OVX group (Table 2).

In the femoral diaphysis of OVX + FRM rats, an increase in maximum load and fracture load (by 4.4% and 4.1%, resp.) and a decrease of displacement for maximum load and displacement for fracture load (by 4.6% and 4.1%, resp.) were noted. The value of Young's modulus was higher (by 22.0%) in OVX + FRM group when compared to the OVX group. The maximum load and the fracture load in tibial metaphysis of OVX + FRM rats increased (by 19.7% and 29.9%, resp.) in comparison with the OVX group. Displacement for maximum load and displacement for fracture load were not changed; however, there was an increase in Young's modulus value (by 19.3%), as compared to OVX group. There was no change in the maximum load applied to the femoral neck when compared to the OVX group (Table 3).

In OVX + FRM rats, in femur, the water content decreased (statistically significant by 15.0%), content of the organic substances increased (statistically significant by 12.2%), while the mineral substances content was not changed, when compared to the OVX group. In tibia, the content of water was lower (statistically significant by 19.8%), while the content of organic and mineral substances was higher (by 3.6% and 10.5%, resp.) in OVX + FRM group than in the OVX rats. The water content decreased (by 16.1%), and an increase of organic (by 7.7%) and mineral fraction content (by 7.7%) in L-4 vertebra in OVX + FRM group when compared to the OVX group was observed. The calcium and phosphorus content in all examined bones was not significantly changed in OVX + FRM rats in comparison with the OVX group (Table 4).

## 4. Discussion

Phytoestrogens can relieve the symptoms of menopause; thus, they are promising substances for preventing bone fractures in women suffering from a postmenopausal osteoporosis. Genistein is the best-studied substance among all phytoestrogens which affect bone mechanical properties. The experiments carried out on laboratory animal models revealed that genistein shows a bone-sparing effect. Wang et al. [31] demonstrated that administration of this isoflavone at doses of 4.5, 9, and 18 mg/kg to ovariectomized rats not only improved the bone mineral density (BMD) of the tibia and femur, but also showed beneficial effect on the bone chemical composition—calcium, phosphorus, and magnesium ions content in tibia was higher in ovariectomized rats treated with genistein than in untreated ones. Bitto et al. [32] described the effect of subcutaneously administered genistein to ovariectomized rats at a dose of 10 mg/kg on BMD and bone mineral content (BMC). Both analyzed parameters were higher than in ovariectomized rats receiving other treatments (alendronate, raloxifene, or 17-*α*-ethinyl oestradiol). Furthermore, the administration of this phytoestrogen improved the breaking strength of femur, and this improvement was also greater when compared to the other examined substances. Miao et al. [33] also highlighted the significance of this isoflavone as a substance improving BMD and bone resistance in ovariectomy-induced osteoporosis. Aside from the experiments for osteoporosis carried out on the laboratory animal model, many clinical trials of the postmenopausal women, that confirm the beneficial effect of genistein on skeletal system, were conducted [34–36].

In spite of a large number of reports regarding genistein, publications about the effects of other phytoestrogens (such as biochanin A, daidzein, or formononetin) on bone microarchitecture, biomechanical properties, and chemical composition are still scarce.

Formononetin is suggested to be a natural selective estrogen receptor modulator (SERM) [37, 38]. SERMs, such as raloxifene, reveal estrogenic activity in bone cells, thus they inhibit osteoporosis development in postmenopausal women, without uterotropic and mammotropic effects [39–41].

The first scientific reports about beneficial effect of formononetin on bone tissue focused on *in vitro* studies of bone cells [15, 22, 42] and on bone histomorphometric parameters of laboratory animals [13, 38]. Ha et al. [13] showed that intraperitoneally administered formononetin at doses of 1 and 10 mg/kg leads to an increase of the trabeculae area in tibia and lumbar vertebra. What is more, Tyagi et al. [38] also described the improvement of the bone microarchitecture by *μ*CT method. The authors noted that in tibia and femur the number and thickness of bone trabeculae increased and the distance between them decreased. These observations coincide with the results obtained in our study (data not shown). Some authors suggest that the examination of bone biomechanical features is essential for confirming beneficial effect of formononetin demonstrated in previous studies [37, 38]. It is widely known that, apart from bone microarchitecture, the chemical composition of bones has an influence on its mechanical properties. Biomechanical features of bones contribute to resistance and susceptibility to fractures, which are the most dangerous symptoms of osteoporosis [43]. Due to the correlation mentioned above, our study, as the first one, focused on the effect of formononetin on chemical composition and mechanical resistance of bones.

Experiment was carried out on the rat model of ovariectomy-induced osteoporosis. Three-month-old female rats were operated—both ovaries were excised, leading to estrogen deficiency and, in consequence, to osteoporotic changes. Formononetin was administered to rats at a dose of 10 mg/kg *po* for 4 weeks. The dose of examined isoflavone was determined on the basis of the literature reports indicating beneficial effect of formononetin on the bone microarchitecture and bone cells *in vitro* [13, 42]. In our experiment, as a positive control, the ovariectomized rats receiving estradiol at a dose of 0.2 mg/kg were used.

Estrogen deficiency induced characteristic changes in ovariectomized rats as follows: an intensified body mass gain, decreased uterus mass, and increased thymus mass. Ovariectomy also induced deteriorations in bone mechanical properties. A decrease of maximum load in femoral diaphysis, tibial metaphysis, and femoral neck, as well as a decrease of fracture load in femoral diaphysis and tibial metaphysis was observed when compared to the sham-operated group.

Young's modulus in femoral diaphysis and tibial metaphysis also was lower than in sham-operated rats. What is more, an increase in displacement for maximum and fracture loads, both in the femoral diaphysis and tibial metaphysis, was noted. Mechanical resistance of skeletal system depends on the mass and shape of bones, as well as on their internal structure and chemical composition. Thus, the impairment of bone biomechanical features in ovariectomized rats can be caused by reduced mass of femur and tibia, as well as alterations in shape–decreased diameter of long bones. Adverse osteoporotic changes in ovariectomized rats were also revealed in histomorphometric parameters, especially in bone trabeculae loss (data not shown). These results indicate that bones in ovariectomized rats are less resistant to fractures and more susceptible to deformations than in sham-operated group.

The lack of estrogen also affected bone chemistry: an increased water and organic content, as well as decreased mineral fraction, was observed. It was previously mentioned that ovariectomy can induce significant increase of water and decrease of mineral fraction content in bones [44]. Calcium content in bones also was lower than in sham-operated animals.

Similar changes in the ovariectomy rat model were previously observed [30, 45–47]. Estradiol supplementation caused withdrawal of some alterations connected with the uterus and body mass. Administration of estradiol prevents deterioration of the bone mechanical features in ovariectomized rats. The maximum load, fracture load, displacements for maximum and fracture loads in femoral diaphysis, and maximum load in femoral neck are practically restored to values for sham-operated group. In the tibial metaphysis, all of the described biomechanical parameters excluding displacement for maximum load were also improved when compared to ovariectomized rats. Thus, bones in ovariectomized rats receiving estradiol are more resistant to fractures and deformations than in ovariectomized rats without supplementation. It is also confirmed by an increased value of Young's modulus. Young's modulus is a parameter describing elasticity and internal rigidity of bones [48]. Beneficial effect of estradiol on skeletal system could be a result of improvement of bone chemistry—level of water and organic fraction decreased and the content of minerals increased in all analyzed bones. Calcium concentration in all examined bones was also slightly improved.

It is worth noting that estradiol causes an increase of the bone trabeculae width in femoral metaphysis and epiphysis (data not shown).

The earlier studies, where the ovariectomized rats treated with estradiol were used as a positive control, also demonstrate that supplementation with this substance shows beneficial effect on the mineralization, microarchitecture, and mechanical parameters of bones [46].

As opposed to the results obtained in estradiol-receiving group, administration of formononetin to ovariectomized rats did not affect the body mass gain and uterus mass. No uterotropic effect after administration of formononetin was observed in other studies, where this isoflavone was administered to ovariectomized rats (at doses of 0.1, 1.0, and 10 mg/kg i.p.) and to immature female rats at a dose of 10 mg/kg *po* [13, 42]. However, Mu et al. indicated that uterotropic effect in ovariectomized mice can be observed, when formononetin is administered in higher doses, that is, 50 and 500 mg/kg [20].

In our study, the beneficial effect of formononetin on the biomechanical parameters in rats with ovariectomy-induced osteoporosis was observed.

In the femoral diaphysis, an increase of both the maximum and fracture loads was observed, while displacement for maximum load and fracture load decreased. The effect of formononetin on described biomechanical features in femoral diaphysis was weaker as compared to ovariectomized rats treated with estradiol. On the contrary, Young's modulus in femoral diaphysis increased, and its value was higher than in the ovariectomized rats receiving estradiol.

In measurements of mechanical features in tibial metaphysis of ovariectomized rats treated with formononetin, an increase of maximum load and fracture load was noted. The examined isoflavone, however, did not affect displacements of both maximum and fracture loads. Beneficial effect of formononetin on tibial metaphysis was lower than in rats receiving estradiol; nevertheless, Young's modulus value, similarly to femoral diaphysis, was higher in ovariectomized rats treated with formononetin than in group administered with estradiol.

Administration of formononetin to ovariectomized rats prevented femur, tibia, and L-4 vertebra mass loss, which occurs in estrogen deficiency conditions. Thus, the improvement of biomechanical features in femoral diaphysis and tibial metaphysis can be a result of the formononetin's ability to obviate bone mass loss in ovariectomized rats.

In contrast to estradiol supplementation, administration of formononetin to ovariectomized rats showed no beneficial effect on mechanical properties in the femoral neck.

Weaker effect of formononetin on biomechanical features of examined bones can be caused by lower affinity of this isoflavone to estrogen receptors when compared to estradiol [13]. Administration of formononetin to ovariectomized rats caused a decrease of water content in all analyzed bones, like in the group treated with estradiol. One of the basic medicines commonly used in the treatment of postmenopausal osteoporosis is alendronate. Its positive effect on bones is also demonstrated by a decrease of water content in ovariectomized rats [44]. In our experiment, a slight increase of mineral content in femur and tibia in ovariectomized rats was observed.

Previous *in vivo* studies conducted by Gautam et al. [42] indicated that formononetin advantageously affects bone mineralization. By using dynamic histomorphometric method, they observed that administration of this isoflavone to rats caused slight, but statistically significant, increase of mineralizing surface of femur.

As opposed to the group supplemented with estradiol, administration of formononetin did not cause a decrease of organic fraction content in femur, tibia, and L-4 vertebra but led to its increase. Most likely, beneficial decline of water content was not completely compensated by meager increase of minerals, which consequently resulted in higher organic substances content in bones of ovariectomized rats receiving formononetin.

An increase of organic fraction content in bones of formononetin-treated ovariectomized rats can be also explained by an enhanced collagen synthesis. *In vitro* studies on osteoblasts [15] and *in vivo *examinations on the rat fracture model [22] indicated that formononetin leads to higher expression of collagen markers.

An above-mentioned increase of Young's modulus value in analyzed bones can be related to the elevated organic fraction content after treatment with examined isoflavone. The effect of formononetin on mineralization processes *in vivo* needs further investigations.

Osteoprotective effect of formononetin on skeletal system of ovariectomized rats may result from the inhibition of bone turnover rate. Tyagi et al. [38] observed a significant decrease of serum osteocalcin and urinary Carboxy-terminal Collagen Crosslinks (CTx) levels in ovariectomized rats, which may imply that formononetin inhibits bone turnover rate which is increased under estrogen deficiency in a characteristic way. These authors also noted that administration of formononetin to ovariectomized rats results in elevated level of OPG genes mRNA and reduced mRNA level of RANKL genes; thus, OPG/RANKL ratio increased, which is an evidence of inhibited osteoclastogenesis. Gautam et al. [42] conducted research on the effects of formononetin on osteoblasts. They noted that formononetin does not affect proliferation of the osteoblasts; however, it induces differentiation of osteoprogenitor cells to mature osteoblasts via p38 MAPK kinase activation.

Beneficial effect of formononetin on bones in ovariectomized rats may also result from antioxidative activity of this isoflavone, which was demonstrated by Mu et al. [20]. These authors carried out an experiment on ovariectomized mice, which were treated with formononetin at doses of 0.05 and 0.5 g/kg. Ovariectomy induced a reduction of superoxide dismutase (SOD), catalase (CAT) and glutathione peroxidase (GSH-Px) activity, and enhancement of lipids peroxidation (LPO). Those results coincide with earlier studies conducted by Muthusami et al. [49]. Formononetin administered by Mu et al. [20] led to an increase of SOD, CAT, and GSH-Px activity and lowered LPO. Free radicals are responsible for osteoblast's apoptosis induction and inhibition of osteoblastogenesis as well as an activation of osteoclasts differentiation via RANKL pathway [50, 51]. For this reason, administration of antioxidants may have beneficial effect on the bone tissue altered by osteoporosis.

Studies conducted by Muhammad et al. [52] on ovariectomy-induced osteopenic rats revealed an improvement of bone microarchitecture as well as an inhibition of further bone tissue loss after vitamin E (which is one of the most powerful antioxidants) administration.

It can be assumed that, due to its antioxidative activity, formononetin may also reveal beneficial effect on osteoporotic bones.

An intake of phytoestrogens is commonly considered as a safe therapy. Despite the fact that information about adverse effects of this treatment is scarce, there are some individual reports revealing side effects of such supplementation. Rachoń et al. indicated that long-term treatment with high doses of equol (one of the most potent phytoestrogens) may result in pathological changes in uterine weight and histological features, and it can also lead to mammotropic effects [53, 54]. There is also a report that another class of phytoestrogens—lignans—may contribute to the risk of prostate cancer among men and collateral cancer among women [55]. What is more, Leclercq et al. [56] noted that phytoestrogens lack the selectivity of action; thus, they may interact with other receptors. All things considered, even though there are only few reports about the adverse effects of phytoestrogens administration, women who take such treatment should inform their physician about being under medical survey.

## 5. Conclusions

To summarize, administration of formononetin to ovariectomized rats shows beneficial effect on biomechanical features and chemical composition of bones; thus, it prevents osteoporotic changes development. Therefore, it can be assumed that this isoflavone may be useful in the prevention and treatment of postmenopausal osteoporosis in women. However, this issue needs further investigations.

## Figures and Tables

**Table 1:** Effects of estradiol and formononetin on the body mass gain and mass of organs in ovariectomized rats.

Parameters	SHAM	OVX	OVX + ES	OVX + FRM
Final body mass (g)	263.3 ± 10.5	272.6 ± 3.6	252.9 ± 7.2^B^	270.0 ± 2.4
Body mass gain after 4 weeks (g)	32.1 ± 2.9	50.6 ± 3.8^AA^	34.1 ± 4.5^B^	49.8 ± 2.2
Uterus mass (g)	0.39 ± 0.01	0.09 ± 0.01^AAA^	0.18 ± 0.01^BBB^	0.09 ± 0.01
Thymus mass (g)	0.39 ± 0.04	0.68 ± 0.03^AAA^	0.62 ± 0.05	0.72 ± 0.03

Results are presented as means ± SEM (*n* = 7). ^AA^ *P* < 0.01,^AAA^ *P* < 0.001—statistically significant differences between the OVX and the SHAM groups; ^B^ *P* < 0.05, ^BBB^ *P* < 0.001—statistically significant differences in comparison with the OVX group.

**Table 2:** Effects of estradiol and formononetin on bone macrometric parameters in ovariectomized rats.

Parameters	SHAM	OVX	OVX + ES	OVX + FRM
Femur				

Mass/body mass (g/kg)	2.78 ± 0.08	2.5 ± 0.02^AA^	2.65 ± 0.03^BB^	2.61 ± 0.07
Length (mm)	35.5 ± 0.3	35.7 ± 0.3	35.7 ± 0.5	35.6 ± 0.2
Diameter (mm)	3.0 ± 0.05	2.84 ± 0.04	2.90 ± 0.05	2.94 ± 0.06

Tibia				

Mass/body mass (g/kg)	2.02 ± 0.08	1.82 ± 0.03^A^	1.89 ± 0.04	1.86 ± 0.02
Length (mm)	37.8 ± 0.6	38.0 ± 0.4	37.9 ± 0.5	38.2 ± 0.4
Diameter (mm)	2.29 ± 0.05	2.12 ± 0.01^A^	2.20 ± 0.06	2.22 ± 0.05

L-4 vertebra				

Mass/body mass (g/kg)	0.71 ± 0.04	0.61 ± 0.01	0.68 ± 0.02	0.67 ± 0.03

Results are presented as means ± SEM (*n* = 7). ^A^ *P* < 0.05, ^AA^ *P* < 0.01—statistically significant differences between the OVX and the SHAM groups; ^BB^ *P* < 0.01—statistically significant differences in comparison with the OVX group.

**Table 3:** Effects of estradiol and formononetin on bone mechanical properties in ovariectomized rats.

Parameter	SHAM	OVX	OVX + ES	OVX + FRM
Femoral diaphysis				

Maximum load (N)	94.0 ± 3.6	88.2 ± 3.5	93.2 ± 5.6	92.1 ± 3.8
Displacement for maximum load (mm)	0.60 ± 0.02	0.65 ± 0.02	0.58 ± 0.01	0.62 ± 0.01
Fracture load (N)	93.9 ± 3.6	84.5 ± 6.0	94.0 ± 8.3	88.0 ± 3.8
Displacement for fracture load (mm)	0.61 ± 0.03	0.72 ± 0.07	0.60 ± 0.09	0.63 ± 0.03
Young's modulus (MPa)	4673.9 ± 474.7	4386.7 ± 384.4	4933.7 ± 333.5	5352.2 ± 463.7

Tibial metaphysis				

Maximum load (N)	114.9 ± 5.1	56.3 ± 7.9^AAA^	75.9 ± 10.0	67.4 ± 5.4
Displacement for maximum load (mm)	0.85 ± 0.07	0.86 ± 0.05	0.90 ± 0.07	0.91 ± 0.06
Fracture load (N)	110.7 ± 3.8	50.4 ± 9.9^AAA^	75.4 ± 9.9^B^	65.5 ± 4.9
Displacement for fracture load (mm)	1.01 ± 0.07	1.16 ± 0.27	1.06 ± 0.22	1.09 ± 0.02
Young's modulus (MPa)	3318.2 ± 957.6	1628.3 ± 449.3^A^	1774.3 ± 492.3	1943.3 ± 228.3

Femoral neck				

Maximum load (N)	93.2 ± 3.1	80.9 ± 6.2	90.2 ± 5.3	78.6 ± 4.8

Results are presented as means ± SEM (*n* = 7). ^A^ *P* < 0.05, ^AAA^ *P* < 0.001—statistically significant differences between the OVX and the SHAM groups; ^B^ *P* < 0.05—statistically significant differences in comparison with the OVX group.

**Table 4:** Effects of estradiol and formononetin on bone mineral, H_2_O, organic compounds, and calcium and phosphorus content in ovariectomized rats.

Parameter	SHAM	OVX	OVX + ES	OVX + FRM
Femur				

H_2_O content (mg/100 mg bone mass)	29.0 ± 0.2	33.4 ± 0.2^AA^	30.7 ± 0.5^B^	28.4 ± 1.3^B^
Organic compounds (mg/100 mg bone mass)	24.7 ± 0.3	28.7 ± 0.6^AAA^	24.0 ± 0.5^BB^	32.2 ± 0.8^B^
Mineral content (mg/100 mg bone mass)	45.3 ± 1.3	38.1 ± 2.7^AA^	44.4 ± 3.5^B^	40.0 ± 2.8
Calcium content (mg/100 mg mineral substances)	39.9 ± 3.4	35.6 ± 5.6	38.7 ± 4.5	36.2 ± 6.0
Phosphorus content (mg/100 mg mineral substances)	28.9 ± 1.4	27.6 ± 2.7	29.8 ± 3.4	27.9 ± 3.9

Tibia				

H_2_O content (mg/100 mg bone mass)	26.6 ± 0.9	30.8 ± 2.1^A^	27.5 ± 0.7^B^	24.7 ± 1.2^BB^
Organic compounds (mg/100 mg bone mass)	28.1 ± 0.4	32.8 ± 0.3^AA^	26.9 ± 1.4^B^	33.9 ± 1.8
Mineral content (mg/100 mg bone mass)	45.5 ± 2.4	37.3 ± 5.9	45.9 ± 1.3^B^	41.2 ± 3.3
Calcium content (mg/100 mg mineral substances)	42.3 ± 4.9	38.4 ± 5.1	43.3 ± 2.3	39.0 ± 6.8
Phosphorus content (mg/100 mg mineral substances)	30.6 ± 1.1	29.2 ± 2.2	29.6 ± 1.8	29.0 ± 2.5

L-4 vertebra				

H_2_O content (mg/100 mg bone mass)	27.6 ± 0.5	32.4 ± 1.7^A^	27.9 ± 1.5	27.2 ± 1.1
Organic compounds (mg/100 mg bone mass)	28.2 ± 0.4	35.3 ± 0.7^AA^	29.1 ± 2.0^B^	38.0 ± 3.5
Mineral content (mg/100 mg bone mass)	44.4 ± 0.3	32.5 ± 3.8^AA^	43.0 ± 0.7^BBB^	35.0 ± 2.7
Calcium content (mg/100 mg mineral substances)	43.7 ± 5.8	39.9 ± 2.6	41.3 ± 1.5	38.3 ± 7.5
Phosphorus content (mg/100 mg mineral substances)	33.7 ± 2.5	31.3 ± 4.3	31.1 ± 2.3	35.0 ± 2.9

Results are presented as means ± SEM (*n* = 7). ^A^ *P* < 0.05, ^AA^ *P* < 0.01, ^AAA^ *P* < 0.001—statistically significant differences between the OVX and the SHAM groups; ^B^ *P* < 0.05,^ BB^ *P* < 0.01, ^BBB^ *P* < 0.001—statistically significant differences in comparison with the OVX group.
